# A discrete event simulation model of clinical and operating room efficiency outcomes of sugammadex versus neostigmine for neuromuscular block reversal in Canada

**DOI:** 10.1186/s12871-016-0281-3

**Published:** 2016-11-16

**Authors:** Ralph P. Insinga, Cédric Joyal, Alexandra Goyette, André Galarneau

**Affiliations:** 1 Department of Predictive & Economic Modeling, Merck & Co., Inc, PO Box 1000, UG1CD-32, North Wales, PA 19454-1099 USA; 2 Department of Health Economics and Observational Research, 16750 Transcanada Highway, Merck Canada, Kirkland, Québec H9H 4M7 Canada; 3 Department of Medical Affairs, 16750 Transcanada Highway, Merck Canada, Kirkland, Québec H9H 4M7 Canada

**Keywords:** Neuromuscular block, Reversal, Sugammadex, Neostigmine, Residual blockade, Operating room, Efficiency

## Abstract

**Background:**

The objective of this analysis is to explore potential impact on operating room (OR) efficiency and incidence of residual neuromuscular blockade (RNMB) with use of sugammadex (Bridion™, Merck & Co., Inc., Kenilworth, NJ USA) versus neostigmine for neuromuscular block reversal in Canada.

**Methods:**

A discrete event simulation (DES) model was developed to compare ORs using either neostigmine or sugammadex for NMB reversal over one month. Selected inputs included OR procedure and turnover times, hospital policies for paid staff overtime and procedural cancellations due to OR time over-run, and reductions in RNMB and associated complications with sugammadex use. Trials show sugammadex’s impact on OR time and RNMB varies by whether full neuromuscular recovery (train-of-four ratio ≥0.9) is verified prior to extubation in the OR. Scenarios were therefore evaluated reflecting varied assumptions for neuromuscular reversal practices.

**Results:**

With use of moderate neuromuscular block, when full neuromuscular recovery is verified prior to extubation (93 procedures performed with sugammadex, 91 with neostigmine), use of sugammadex versus neostigmine avoided 2.4 procedural cancellations due to OR time over-run and 33.5 h of paid staff overtime, while saving an average of 62 min per OR day. No difference was observed between comparators for these endpoints in the scenario when full neuromuscular recovery was not verified prior to extubation, however, per procedure risk of RNMB at extubation was reduced from 60% to 4% (reflecting 51 cases prevented), with associated reductions in risks of hypoxemia (12 cases avoided) and upper airway obstruction (23 cases avoided).

Sugammadex impact in reversing deep neuromuscular block was evaluated in an exploratory analysis. When it was hypothetically assumed that 30 min of OR time were saved per procedure, the number of paid hours of staff over-time dropped from 84.1 to 32.0, with a 93% reduction in the per patient risk of residual blockade.

**Conclusions:**

In clinical practice within Canada, for the majority of patients currently managed with moderate neuromuscular block, the principal impact of substituting sugammadex for neostigmine is likely to be a reduction in the risk of residual blockade and associated complications. For patients maintained at a deep level of block to the end of the procedure, sugammadex is likely to both enhance OR efficiency and reduce residual block complications.

**Electronic supplementary material:**

The online version of this article (doi:10.1186/s12871-016-0281-3) contains supplementary material, which is available to authorized users.

## Background

Neuromuscular blocking agents (NMBAs) are often administered during surgical procedures to provide muscle relaxation, and to prevent patient movement, which may increase the risk of surgical complications. When neuromuscular block no longer needs to be maintained, patients may either be allowed to spontaneously recover neuromuscular function or be administered a reversal agent for more rapid recovery. The acetylcholinesterase inhibitor neostigmine is commonly used for reversal of moderate neuromuscular blockade (e.g., when at least the second twitch [T_2_] of a train-of-four stimulation is present). Due to the occurrence of muscarinic side effects with neostigmine such as nausea, vomiting and bradycardia, it is typically co-administered with an anti-muscarinic agent such as atropine or glycopyrrolate [1]. Recovery of neuromuscular function via either spontaneous reversal or use of neostigmine is neither rapid nor of predictable duration [2, 3]. Patients may therefore be inadvertently extubated while still experiencing residual neuromuscular paralysis (residual neuromuscular blockade), with accompanying respiratory and muscular complications [4].

Sugammadex (Bridion™, Merck & Co., Inc., Kenilworth, NJ USA, sponsor of the present analysis), a modified gamma-cyclodextrin, is a more recently developed reversal agent, recently approved for commercialization in Canada (and also licensed in the United States and European Union) to reverse neuromuscular blockade induced by the NMBAs rocuronium or vecuronium [5]. In clinical trials, sugammadex has been shown to produce much more rapid and predictable reversal of neuromuscular block than neostigmine, in the absence of anti-muscarinic side effects and, in trials where quantitative neuromuscular monitoring was not required, a steep reduction in the incidence of residual neuromuscular blockade (RNMB) [3, 5–8]. In addition, sugammadex is efficacious in rapidly reversing deep neuromuscular block (i.e., at re-appearance of 1–2 post-tetanic counts), [2, 9, 10] whereas acetylcholinesterase inhibitors such as neostigmine cannot adequately reverse deep levels of blockade because they reach a “ceiling” effect in which the increase in acetylcholine concentration is insufficient to displace enough NMBA molecules to reverse neuromuscular block [10, 11].

The objective of the present analysis is to explore potential impact on operating room (OR) efficiency and incidence of RNMB with use of sugammadex versus neostigmine for routine reversal of neuromuscular blockade in Canada. This paper describes a discrete event simulation (DES) model developed for this purpose. Outcomes explored include selected clinical events associated with residual neuromuscular blockade and OR efficiency-related measures.

## Methods

Ethical committee approval was not required for the present study as modeling was based on secondary data sources and human or animal subjects were not enrolled.

### Perspective

The model perspective is that of a Canadian hospital. An OR schedule and patient clinical outcomes associated with neuromuscular blockade were simulated over a 1 month period (21 working days) for ORs utilizing either sugammadex or neostigmine for neuromuscular block reversal.

### Treatment comparators

The primary analyses evaluate reversal of moderate neuromuscular blockade, with sugammadex (2 mg/kg) compared to neostigmine (50 μg/kg) plus glycopyrrolate (10 μg/kg). Reversal of deep neuromuscular block is evaluated as an exploratory analysis utilizing a 4 mg/kg dose of sugammadex. Neuromuscular block was assumed to be maintained with either rocuronium or vecuronium (though in Canada vecuronium is not utilized), for which sugammadex is indicated for reversal.

Acetylcholinesterase inhibitors such as neostigmine cannot adequately reverse deep levels of blockade [10, 11]. In clinical practice, for procedures utilizing deep block, the level of neuromuscular blockade is generally allowed to fade to moderate, prior to administering neostigmine. This scenario was modeled as an exploratory analysis as there has not been a corresponding clinical trial of sugammadex to date in which all patients in the neostigmine arm were administered deep block which was allowed to fade to moderate prior to reversal agent administration, and hypothetical values for potential time savings with sugammadex use were therefore evaluated.

### Structure

The DES model was developed in part with Arena Version 14.7 software (Rockwell Automation, Milwaukee, WI), with a Microsoft Excel 2010 (Redmond, WA) interface used for input entry and output reporting. The model compares ORs using either neostigmine or sugammadex for NMB reversal, for the same simulated schedule of procedures performed daily on 21 working days over 1 month. The impact of using sugammadex compared to neostigmine on OR procedure time (time from OR admission to OR discharge) within clinical trials has varied according to how neuromuscular recovery and extubation have been managed [12–16]. Because neuromuscular recovery practices may vary by institution, the model therefore evaluates a variety of different scenarios for the proportion of patients (0%, 5%, 10%, 25%, 50%, 75%, 100%) verified to have full neuromuscular recovery (TOF ratio ≥ 0.9) prior to extubation in the OR.

**Fig. 1:**
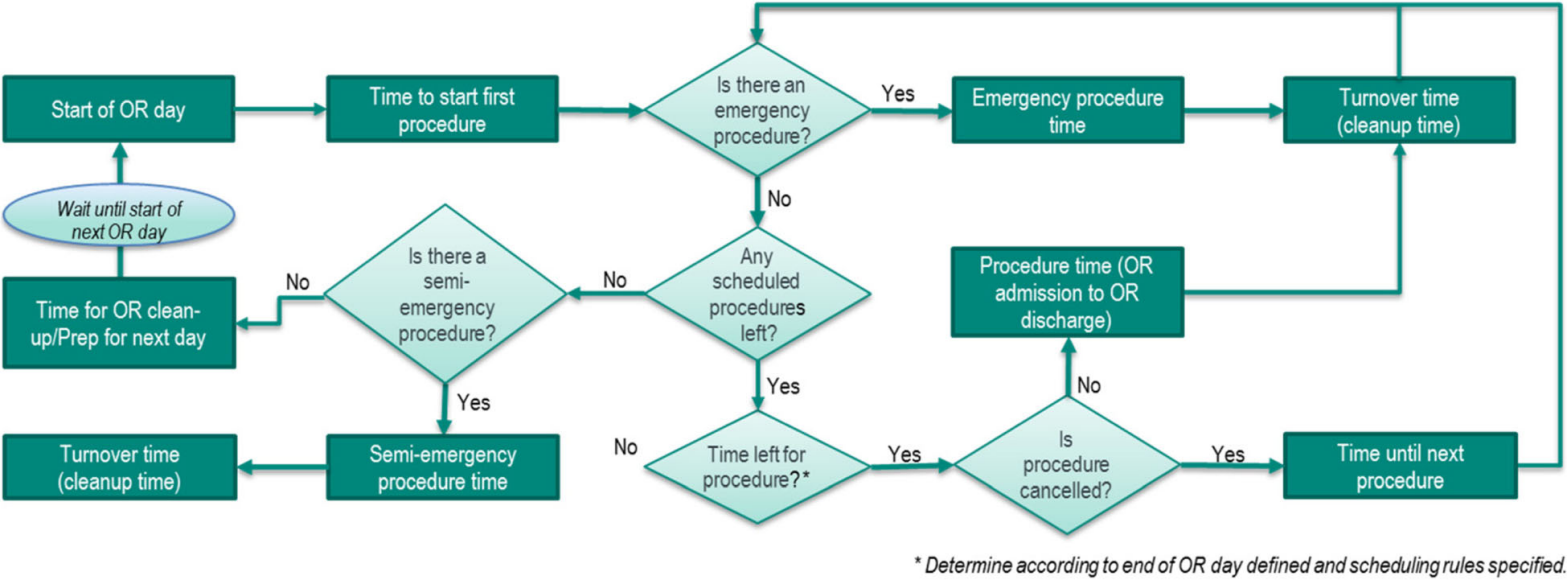
Diagram of operating room day and procedural flow

**Fig. 2:**
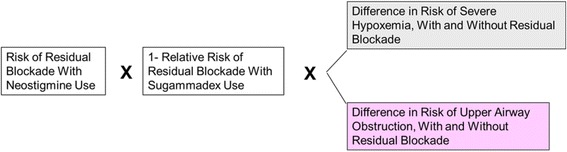
Model structure for reduction in risk of residual neuromuscular blockade and associated complications

Random durations are generated for the OR procedures utilizing the assumed mean duration for each procedure and its statistical distribution. Random values are generated similarly for other parameters influencing procedural flow, including time from the start of the OR day to initiation of the 1^st^ procedure, turnover times between procedures, frequency of emergency procedures (immediately inserted into the OR schedule bumping the next scheduled procedure), frequency of semi-emergency procedures (added on at the end of the OR day), probability and impact of procedural cancellations due to reasons other than OR time over-run and time for staff clean-up of the OR at the end of the day. Additional model parameters include hospital policies for procedural cancellation when OR time over-run occurs, impact of sugammadex on OR time per procedure compared to neostigmine, risk of residual neuromuscular blockade and its clinical sequelae with neostigmine use and risk reductions with sugammadex and staff eligible for OR overtime pay. An overview of the model structure is shown in Fig. 1 for OR procedural flow and in Fig. 2 for residual neuromuscular blockade and associated complications.

The aforementioned parameters are used to estimate sugammadex’s impact versus neostigmine on OR times and procedural flow, staff overtime, procedural cancellation and risk of residual neuromuscular blockade and associated complications.

### Inputs

A series of systematic literature searches of the US National Library of Medicine’s PubMed database (http://www.ncbi.nlm.nih.gov/pubmed/) were conducted to obtain data values for various model parameters. In addition, to the PubMed database, the Cochrane Library and University of York and Centre for Reviews and Dissemination (CRD) databases were also searched. Reference lists within publications of potential relevance were further searched to identify additional studies. Proprietary informational sources providing relevant data were also reviewed. For conciseness, the specific PubMed literature search strategies for each model input are described in detail in Additional file 1. PubMed searches were conducted for literature published up to August 21, 2015.

For operating room scheduling and staffing parameters (e.g., time to start of OR day, turnover times, time for OR clean-up, procedure duration, OR staff eligible for over-time pay), values would expected to be highly variable across operating rooms and institutions. With respect to scheduling, available publications reporting data often reflect examples where efficiency was less than desired and improvements were sought. Rather than conducting a formal meta-analysis across publications, data from available sources deemed as reasonable and credible by OR experts were included within the model and values were varied in reported sensitivity analyses.

In clinical practice, parameters for the OR day and procedural flow vary by hospital and OR. Illustrative examples of parameter values for these variables were gathered from the literature or based on assumption as described in Table 1. Estimation methods for other individual model parameters are described in greater detail within this section.

**Table 1:** Base case model inputs

Parameter	Default value^a^	Source
Time Horizon	1 month (21 working days)	Assumption
Operating Room		
Start of OR day	7:30 AM	Assumption
End of OR day	4:00 PM	Assumption
Time from start of OR day to OR admission of first patient	10 min	[36]
Time between procedures (turnover time)	35.6 min	[37]
Time for OR clean-up/prep for next day	15 min	Assumption
Procedure		
Number of procedures per day	5	Assumption
Mean time per procedure with neostigmine use	72.9 min	Analysis of RECITE Canada data (data on file)
Probability of cancellation of schedule procedure (unrelated to OR time over-run)	10.7%	[38]
Is next procedure moved up when cancellation occurs?	No	Assumption
Can a procedure be cancelled because there is not enough OR time available?	Yes	Assumption
Cancellation policy	No procedures may begin after end of OR day	Assumption
Probability of emergency procedure insertion	0%	Assumption
Probability of semi-emergency procedure insertion	0%	Assumption
Residual Neuromuscular Block		
Risk of residual block at extubation with neostigmine use	60.0%	[6, 19, 20]
Absolute excess risk of hypoxemia with residual block	24.5%	[33]
Absolute excess risk of upper airway obstruction with residual block	44.2%	[6, 32]
Impact of Sugammadex vs. Neostigmine		
Reduction in time from patient OR admission to OR discharge		
All patients verified to have full neuromuscular recovery (TOF ratio ≥0.9) prior to extubation in the OR	14 min	Grintescu et al. 2009 [16]; P318 2010 [13]
No patients verified to have full neuromuscular recovery (TOF ratio ≥0.9) prior to extubation in the OR	0 min	P334 2009 [12]; P07981 2013 [14]; P07038 2014 [15]
Reduction in risk of residual neuromuscular blockade at extubation, among patients not verified to have full neuromuscular recovery (TOF ratio ≥0.9) prior to extubation	93%	[6]
OR Staff Eligible for Overtime Pay		
Registered nurses	3 present	Assumption
Respiratory therapist	1 present	Assumption
Nurse aide	1 present	Assumption
Overtime pay policy	30 min increments	Assumption

*OR* Operating room

^a^Assumed Arena probability distributions [Mean time per procedure with neostigmine use (LOGN 72.9,29.2); Turnover time (10+EXPO{25.6}); Time to OR admission of ^1st^ patient (DISC{0.5,5,1,15});OR clean-up time (TRIA{7.5,15,22.5})]

#### Impact of sugammadex vs. neostigmine on OR procedure time

Published clinical trials comparing sugammadex to neostigmine have typically reported information on time from administration of the reversal agent to full patient neuromuscular recovery to a TOF ratio of ≥ 0.9, measured via quantitative neuromuscular monitoring [17]. This is the preferred endpoint for evaluating sugammadex’s efficacy with respect to accelerating neuromuscular recovery, consistent with the direct impact of usage. However, of interest with respect to the present modeling, is the impact of accelerated neuromuscular recovery on the downstream endpoint of patient time in the OR. Comparisons between sugammadex and neostigmine of time from reversal agent administration to full neuromuscular recovery often do not well correlate with impact on overall OR time for several reasons.

First, in clinical practice, patients are often discharged from the OR prior to achieving full neuromuscular recovery, while residual neuromuscular blockade (TOF ratio < 0.9) is present [4, 18]. Second, even where patients have full neuromuscular recovery in the OR, that may not be a rate-limiting step to OR discharge if other necessary activities (e.g., wound suturing, medical equipment removal) occur during this time period currently with use of neostigmine, the time for which cannot be fully eliminated with accelerated neuromuscular recovery. Third, in clinical practice, recognizing the much shorter time to full neuromuscular recovery with sugammadex versus neostigmine, [17] the products may be administered at different time points within a procedure (neostigmine earlier due to longer lead-time needed for an effect and sugammadex later due to very rapid effect in reversing moderate block, and sugammadex earlier and neostigmine later in procedures ending in deep block). As a result, the shortened reversal time with sugammadex would not translate to a commensurate reduction in time to OR discharge. Thus, neuromuscular recovery time is likely to correlate least with patient time in the OR in procedures where there is not full neuromuscular recovery (TOF ratio ≥ 0.9) prior to extubation in the OR, or where deep block is maintained through the end of the procedure, with the potential for a somewhat greater correlation in procedures utilizing a moderate level of block where patients are maintained in the OR through full neuromuscular recovery.

Data were therefore analyzed from available clinical trials reporting time from the endpoint of patient OR admission to OR discharge for subjects randomized to receive sugammadex or neostigmine (Table 2). This endpoint directly corresponds to patient time spent in the OR as evaluated within the DES model. Results were stratified by whether patients in the trial were verified to have full neuromuscular recovery (TOF ratio ≥ 0.9) prior to extubation in the OR, as the impact of sugammadex on OR time has been found to vary according to how neuromuscular recovery is managed. Specifically, larger and statistically significant OR time savings with sugammadex use have been observed in trials where all patients were “verified” to have full neuromuscular recovery (train-of-four [TOF] ratio ≥ 0.9) prior to extubation via quantitative neuromuscular monitoring, whereas time savings have either not been observed or not achieved statistical significance in trials where verification of full neuromuscular recovery was not required [12–16]. Available data were insufficient for further stratification by use of rocuronium vs. vecuronium as the NMBA, or depth of neuromuscular block throughout the procedure or at reversal. Results across trials within each stratified group were pooled via a random effects meta-analysis.

**Table 2:** Sugammadex impact vs. neostigmine on time from patient OR admission to OR discharge, per procedure

A. Trials Requiring Verification^a^ of Full Neuromuscular Recovery (TOF ratio ≥ 0.9) Prior to Extubation in the OR						
*Sugammadex Arm*		*Neostigmine Arm*				
N	Minutes from OR admission to discharge	N	Minutes from OR admission to discharge	SugammadexTime Savings	*P*-value	Source
17	64	17	80	16	0.04	[16]
66 ^b^	158	64	169	11	0.23	[13]
83		81		14	0.02	Meta-analysis
B. Trials Not Requiring Verification of Full Neuromuscular Recovery Prior to Extubation in the OR						
*Sugammadex Arm*		*Neostigmine Arm*				
N	Minutes from OR admission to discharge	N	Minutes from OR admission to discharge	SugammadexTime Savings	*P*-value	Source
48	183	46	167	−16	0.22	[12]
290	167	315	167	0	NA	[15]
74 ^b^	242	77	253	11	0.40	[14]
412		438		−1	0.89	Meta-analysis

*NA* Not applicable *OR* Operating room, *TOF* Train-of-four

^a^Verification of full neuromuscular recovery (TOF ratio ≥ 0.9) based on quantitative neuromuscular monitoring

^b^Numbers below this row reflect a pooling of data via meta-analysis

#### Risk of residual blockade at extubation and associated efficacy of sugammadex

A statistically significant reduction in the incidence of residual neuromuscular blockade at extubation with use of sugammadex versus neostigmine has been demonstrated in a randomized clinical trial where patients were not required to have verification of full neuromuscular recovery prior to extubation in the OR [6]. As additional studies have also reported a risk of residual blockade (TOF < 0.9) at extubation with neostigmine use, a literature search was conducted to estimate the background risk of residual blockade.

Studies describing the risk of residual blockade at extubation exclusively with rocuronium or vecuronium and neostigmine use were gathered from the literature based on two review articles, [4, 18] and a search of the PubMed database using terms of “residual blockade”, “residual block” or “curarization” for studies published from January 1, 2008 (updating from the literature canvassed in the review articles) up to July 10, 2015. Studies using quantitative neuromuscular monitoring to determine the time point for extubation were excluded. Three studies meeting review eligibility criteria were identified and meta-analyzed [6, 19, 20]. Rocuronium was used within each of the studies, and corresponding data were not found for patients receiving vecuronium. However, vecuronium is not utilized within Canada. The resultant average risk of residual block at extubation with neostigmine use was estimated to be 60% when patients are not required to have verification of full neuromuscular recovery (TOF ratio ≤ 0.9) prior to extubation in the OR. Additional details on the estimation are provided in Additional file 1.

The efficacy of sugammadex in preventing residual blockade, specified as a reduction in the risk of residual blockade at extubation with sugammadex usage (93% reduction) compared to neostigmine, was estimated based on data from the aforementioned randomized trial [6].

#### Risk of clinical sequelae of residual blockade

Clinical sequelae of residual blockade may include post-operative aspiration, hypoxemia, muscle weakness and upper airway obstruction.

In the case of aspiration, aspiration pneumonitis (and atelectasis) have been reported as potential outcomes of residual blockade [4, 21]. However, an elevated risk of their occurrence has been observed with long-acting NMBAs, but not with the intermediate-acting NMBAs modeled in this analysis [22] and, to date, a reduction in their incidence with sugammadex use has not been found [23, 24]. Therefore, these clinical events were not modeled as residual blockade outcomes. Uncomplicated aspiration, occurring to the level of, or above, the vocal cords has been reported in volunteer studies with TOF ratios < 0.9, but not below the vocal cords. Due to the rare documentation of aspiration during the post-extubation and recovery periods in general anesthetic settings, [25–27] and likely silent nature of events with lack of clinical management, aspiration was not modeled within the DES.

Muscle weakness, which may be characterized by general weakness, as well as difficulty with speaking and vision, is not uncommon post-operatively [21, 28], and occurs with greater frequency among patients with residual neuromuscular blockade [28, 29]. However, no studies could be identified documenting an excess cost associated with post-operative muscle weakness, nor did clinician consultation suggest this to be a major contributor to resource use. Therefore muscle weakness was also not modeled within the DES.

Published literature were available from which to construct estimates of the excess risk of hypoxemia and upper airway obstruction associated with the occurrence of residual neuromuscular blockade as reported in Table 1. As the estimation methods are fairly detailed, the derivation of these values is described in Additional file 1.

#### Reversal agent resource use

Consistent with prior Canadian health technology assessments (HTAs) and a UK National Institute for Health Research (NIHR) HTA, an average patient weight of 75 kg was assumed, with vial wastage [30]. Consistent with clinical trials evaluating sugammadex, it is assumed that a 50 μg/kg dose of neostigmine is administered, in combination with a 10 μg/kg dose of glycopyrrolate. For sugammadex, it is assumed that doses of 2 mg/kg and 4 mg/kg are used to reverse moderate and deep block, respectively.

#### Operating room overtime resource use

When OR procedures run over the regular OR day, it is assumed that, among the OR staff, registered nurses, respiratory therapists and nurse aides are eligible for overtime pay. Over-time is assumed to be paid in 30 min increments, rounded up to the nearest half hour. It is assumed that 3 registered nurses, one nurse aide and one respiratory therapist are present in a given OR.

### Outputs

Model outputs for an OR, reported over a one month time horizon with respect to use of sugammadex versus neostigmine, include the number of procedures performed, procedural cancellations due to regular OR day over-run, hours of OR staff overtime and cases and complications of residual blockade avoided. Other primary outputs include the number of OR minutes saved per day, % of days all procedures are completed within the regular OR day and reduction in risk of RNMB. Each set of outputs is reported under various scenarios for the % of patients verified to have full neuromuscular recovery (TOF ratio ≥ 0.9) prior to extubation in the OR.

### Sensitivity analyses

Illustrative sensitivity analyses are reported varying the assumed policy for cancelling a procedure due to lack of OR time (cancel if < 50% of procedure can be completed within the regular OR day, never cancel), whether the next procedure is moved up when cancelling (fully move up next procedure), the proportion of procedures in OR which are emergency procedures (15%) and at 95% confidence interval values for sugammadex OR time savings per procedure versus neostigmine.

### Exploratory analysis

An exploratory analysis is conducted evaluating results for the 4 mg/kg dose of sugammadex in reversing patients who are maintained at a deep level of neuromuscular block until the end of the procedure. Sugammadex is efficacious in rapidly reversing deep neuromuscular block (i.e., at re-appearance of 1–2 post-tetanic counts), [2, 10] whereas acetylcholinesterase inhibitors such as neostigmine cannot adequately reverse deep levels of blockade because they reach a “ceiling” in which the increase in acetylcholine concentration is insufficient to displace enough NMBA molecules to reverse neuromuscular block [10, 11]. For these patients in clinical practice, anesthesiologists wait until the depth of block has faded to a moderate level after the completion of the procedure before administering neostigmine. There has not been a corresponding clinical trial of sugammadex to date in which all patients in the neostigmine arm were administered deep block through the end of the procedure, which was allowed to fade to moderate block prior to reversal agent administration. Therefore, hypothetical OR time savings per procedure with sugammadex use of 15, 30, 45 and 60 min were explored.

Because the anesthesiologist must wait to reverse with neostigmine until the depth of block has faded from deep to moderate, it was implicitly assumed based on expert feedback that the procedures would be longer than in the base case when moderate block is used. To achieve an OR schedule of identical average expected duration as in the base case, it was assumed that 3 procedures of 145 min each, utilizing deep block to the end of the procedure, would be scheduled per OR day.

### Simulation

To allow for variability within the OR schedule across OR days, procedure times were drawn from a lognormal distribution around mean values, with discrete, exponential and triangular distributions within ARENA used to draw values for time to first procedure start, turnover times and time for OR clean-up/next day prep, respectively. As point estimate values are varied within the model, a fixed “variability ratio” was chosen for each of the distributions, so that the standard deviation was adjusted according to the mean value entered. The model was run with 50 replications within each analysis, representing 1050 simulated OR days, upon which results converged to stable values.

## Results

### Primary analyses

Comparison of sugammadex and neostigmine in the reversal of moderate neuromuscular block with respect to OR efficiency and clinical outcomes in an OR over a 1 month period is reported in Table 3. The estimated average number of OR minutes saved per day with sugammadex use varies from 0 to 62 min as the percentage of patients verified to have full neuromuscular recovery (TOF ratio ≥ 0.9) prior to extubation in the OR is varied between 0 and 100%. Correspondingly, the percent of days all procedures are completed within the regular OR day with sugammadex use varies from 40.6% (equivalent to with neostigmine use) up to 72.7%, when the percentage of patients verified to have full recovery in the OR is varied from 0 to 100%. Depending on the proportion of patients verified to have full recovery in the OR, the number of procedures performed over 1 month may be increased (range of 0 to 2.2 additional procedures), and number of procedures cancelled due to lack of OR time (range of 0 to 2.4 cancelled procedures avoided) and paid hours of staff over-time decreased with sugammadex use (range of 0 to 33.5 fewer hours).

**Table 3:** Comparison of sugammadex and neostigmine on OR efficiency and clinical outcomes in an OR over 1 month in the reversal of moderate neuromuscular block

Outcome Measure	Neostigmine	Sugammadex (2 mg/kg)						
		% of patients verified to have full neuromuscular recovery (TOF ratio ≥ 0.9) prior to extubation						
		0%	5%	10%	25%	50%	75%	100%
OR efficiency outcomes								
Number of OR minutes saved per day	–	0	3	6	15	31	46	62
% of days all procedures are completed within the regular OR day	40.6%	40.6%	40.8%	42.7%	49.0%	58.0%	65.0%	72.7%
Number of procedures performed	90.8	90.8	90.9	91.0	91.6	92.5	92.9	93.0
Procedures cancelled due to lack of OR time	3.5	3.5	3.4	3.3	2.8	1.9	1.4	1.1
Procedures cancelled for other reasons	10.7	10.7	10.7	10.8	10.7	10.6	10.8	11.0
Paid hours of staff over-time	57.8	57.8	57.4	54.9	47.9	38.9	31.9	24.3
Clinical outcomes								
Cases of residual blockade avoided	–	51	48	46	38	25	13	0
Hypoxemia cases avoided^a^	–	12	12	11	9	6	3	0
Upper airway obstruction cases avoided^a^	–	23	21	20	17	11	6	0
Absolute reduction in risk of residual blockade, per patient	–	56%	53%	50%	42%	28%	14%	0%

*OR* Operating room

^a^Includes both cases which are and are not clinically diagnosed and managed

The impact of sugammadex upon clinical outcomes of RNMB exhibits an opposite trend with respect to neuromuscular recovery practices. The number of cases of residual blockade avoided with sugammadex decreases from 51 to 0 as the percent of patients verified to have full recovery in the OR increases from 0 to 100%, with an absolute reduction in the risk of residual blockade per patient ranging from 56 to 0%. In the scenario where 0% of patients were verified to have full neuromuscular recovery prior to extubation, the numbers needed to treat (NNT) to prevent a case of residual blockade, hypoxemia and upper airway obstruction were 1.8, 7.6 and 3.9, respectively. The NNTs increase as a higher % of patients are verified to have full neuromuscular recovery.

### Sensitivity analyses

Prior work has found OR resource use and costs with sugammadex use to be much less sensitive to offsets associated with residual blockade as compared to OR staff time/over-time [31]. It was therefore elected for illustrative purposes to focus the sensitivity analyses upon the scenario where 100% of patients are verified to have full neuromuscular recovery (TOF ratio ≥ 0.9) in the OR prior to extubation, as OR staff over-time is most influential within this scenario (Table 4).

**Table 4:** Sensitivity Analyses - For scenario where 100% of patients are verified to have full neuromuscular recovery (TOF ratio ≥ 0.9) prior to extubation in the OR

	Number of OR minutes saved per day	% of days all procedures are completed within the regular OR day	Procedures cancelled due to lack of OR time	Paid hours of staff over-time
Primary Analysis^a^				
Neostigmine	–	40.6%	3.5	57.8
Sugammadex	62	72.7%	1.1	24.3
Cancel if < 50% of procedures can be completed within the regular OR day				
Neostigmine	–	39.5%	6.8	36.0
Sugammadex	62	73.7%	1.9	16.5
Never cancel a procedure due to lack of OR time				
Neostigmine	–	40.4%	0.0	92.0
Sugammadex	63	74.3%	0.0	31.4
Fully move up next procedure if a cancellation occurs				
Neostigmine	–	63.2%	2.4	32.3
Sugammadex	62	85.2%	0.7	12.9
Assume 15% of procedures are emergency cases				
Neostigmine	–	19.8%	16.1	108.5
Sugammadex	57	45.0%	9.6	66.6
Sugammadex OR time saved at lower bound of 95% CI in trials (2 min)				
Neostigmine	–	40.6%	3.5	57.8
Sugammadex	9	44.9%	2.8	54.0
Sugammadex OR time saved at lower bound of 95% CI in trials (26 min)				
Neostigmine	–	40.6%	3.5	57.8
Sugammadex	116	92.2%	0.3	5.8

*CI* Confidence interval *OR* Operating room

^a^In the primary analysis, procedures are cancelled if they cannot begin within the regular OR day, when a procedure is cancelled for any reason, the next procedure is not moved up, and no emergency cases occur

In the primary analyses, it is assumed that no procedures may begin after the end of the regular OR day and are otherwise cancelled. When this is modified to assume that cancellation occurs if <50% of the anticipated duration of a procedure may be completed within the regular OR day (a relatively lower threshold for cancelling), the number of procedural cancellations avoided with sugammadex use increases, with a smaller absolute reduction in staff over-time hours. In contrast, if it is assumed that a procedure can never be cancelled due to a lack of OR time, the reduction in paid hours of staff over-time with sugammadex use nearly doubles as compared to within the primary analyses.

If it is assumed that 15% of procedures are emergency cases (as opposed to 0% in the primary analyses), which bump scheduled cases, the number of procedures cancelled due to lack of OR time and paid hours of staff over-time increase dramatically, with relatively larger absolute reductions in these outcomes with sugammadex use as compared to in the primary analyses.

Results were also sensitive to assumed OR time savings with sugammadex versus neostigmine, when varied across values for the 95% confidence interval for this parameter (2 to 26 min) as observed within pooled clinical trial data.

### Exploratory analyses

In the exploratory analyses (Table 5), it is assumed that patients are maintained at a deep level of neuromuscular block through the end of the procedure and reversed with neostigmine or a 4 mg/kg dose of sugammadex. As described in the Methods section, as the amount of time saved with sugammadex versus neostigmine in clinical practice in these patients is unknown, hypothetical time savings of 15, 30, 45 and 60 min per procedure are explored. When the amount of OR time saved is fixed, the percentage of patients verified to have full neuromuscular recovery in the OR no longer influences sugammadex’s impact upon OR time within the analysis. For illustrative purposes, it was therefore elected to conduct the exploratory analysis for the scenario where 0% of patients are verified to have full neuromuscular recovery in the OR, as results would be very similar for the other scenarios, with the exception of the incidence of clinical outcomes of residual blockade.

**Table 5:** Exploratory analyses - Comparison of sugammadex and neostigmine on OR efficiency and clinical outcomes in an OR over 1 month when deep block is maintained to the end of all procedures [0% of patients verified to have full neuromuscular recovery (TOF ratio ≥ 0.9) prior to extubation]

Outcome Measure	Neostigmine	Sugammadex (4 mg/kg)			
		Minutes of OR time saved per procedure			
		15	30	45	60
OR efficiency outcomes					
Number of OR minutes saved per day	–	39	79	118	158
% of days all procedures are completed within the regular OR day	46.7%	61.9%	77.1%	86.9%	91.4%
Number of procedures performed	54.5	54.7	55.1	55.1	55.2
Procedures cancelled due to lack of OR time	1.2	0.7	0.4	0.4	0.3
Procedures cancelled for other reasons	7.3	7.6	7.6	7.6	7.6
Paid hours of staff over-time	84.1	54.9	32.0	18.3	11.5
Clinical outcomes					
Cases of residual blockade avoided	–	30	30	30	30
Hypoxemia cases avoided^a^	–	7	7	7	7
Upper airway obstruction cases avoided^a^	–	13	13	13	13
Absolute reduction in risk of residual blockade, per patient	–	56%	56%	56%	56%

*OR* Operating room

^a^Includes both cases which are and are not clinically diagnosed and managed

In the deep block analysis, as the number of minutes of OR time saved per procedure with sugammadex increases, the number of procedures cancelled due to a lack of OR time decreases, along with paid hours of staff over-time. For instance, the number of paid hours of staff over-time drops from 84.1 to 32.0, when 30 min of OR time are saved per procedure. The number of paid hours of staff over-time with neostigmine use is greater than in the model base case due to the impact of the assumed increased variability in the duration of the procedures for the longer deep block procedures as compared to the shorter moderate block procedures.

## Discussion

This analysis has shown that, depending upon the neuromuscular management and extubation practices at a given hospital, sugammadex can potentially reduce the risk of RNMB and/or enhance operating room efficiency.

For procedures ending with moderate neuromuscular block, when patients are not verified to have full neuromuscular recovery (TOF ratio ≥ 0.9) prior to extubation in the OR, use of neostigmine results in a high incidence of RNMB [6], with associated clinical complications of upper airway obstruction and hypoxemia [32, 33]. To avoid these risks of respiratory complications, if patients administered neostigmine are maintained in the OR until full neuromuscular recovery is verified and they may be safely extubated, additional time is expended within the OR [13, 16]. Sugammadex can ameliorate this trade-off between OR efficiency and the occurrence of residual neuromuscular block by substantially accelerating the time to complete neuromuscular recovery [17] and safe extubation.

When patients are maintained with deep neuromuscular block to the end of the procedure, sugammadex is very likely to save time in the OR compared to neostigmine use, with reductions in the risk of RNMB dependent upon whether patients are verified to have full neuromuscular recovery (TOF ratio ≥ 0.9) prior to extubation in the OR.

In clinical practice, patients currently are rarely verified to have full neuromuscular recovery (TOF ratio ≥ 0.9) prior to extubation in the OR as evidenced by low use of quantitative neuromuscular monitoring [34, 35] and high incidences of RNMB with neostigmine use [6, 19, 20]. Thus, for the majority of patients currently managed with moderate neuromuscular block in Canada, the principal impact of the substitution of sugammadex for neostigmine is likely to be a reduction in the risk of residual blockade and associated complications. Under these conditions, for patients managed with deep neuromuscular block through the end of the procedure, sugammadex is expected to *both* reduce OR procedure times and complications associated with residual blockade relative to use of neostigmine.

In interpreting the magnitude of OR time saved, avoided procedural cancellations, avoided staff overtime and avoided residual block, where applicable, within the present analysis, a number of caveats should be kept in mind. First, given the running time of the DES model to complete iterations for the base case, it was prohibitive to run a formal probabilistic sensitivity analysis (PSA) across model parameters. However, most of the model parameters for which results are most sensitive, as described in the Results section, are based on different scenario-based assumptions for quantitative neuromuscular monitoring, hospital cancellation and emergency procedure policies. These types of parameters are best explored in the one-way sensitivity analyses currently performed within the analysis, as they are not amenable to PSA.

Second, for illustrative purposes, it was assumed that all procedures within the OR day used NMBAs of rocuronium or vecuronium, and neostigmine or sugammadex for neuromuscular reversal. This assumption enables full evaluation of the potential for sugammadex to impact OR time-related outcomes. In clinical practice, however, there may be variation across procedures occurring within a given OR on a particular day in terms of whether NMBAs are used and, if so, whether neuromuscular block is reversed using a pharmaceutical agent, or allowed to spontaneously reverse. In ORs where this variation occurs, the total potential impact of sugammadex on outcomes related to OR time savings and residual block avoidance would be lessened. Also, the primary analysis has modeled 5 short procedures of 72.9 min each. For ORs where 2–3 longer procedures are performed within a given day, all else equal, the potential OR time savings and number of residual blockade cases prevented by sugammadex are also likely to be relatively less.

However, the resource use impact of OR time savings within the present analysis, which was limited to those staff members earning over-time pay due to OR time over-run, is likely to only partially account for the benefits to a hospital. One could also consider the intangible value of time saved for salaried OR staff members who are ineligible for over-time pay (e.g., surgeon and anesthesiologist) as well as non-overtime minutes saved for all OR staff members. Furthermore, when cancelled procedures are avoided, there are potentially impacts for the hospital and patient related to rescheduling, re-preparation for the procedure and patient time/work loss. Finally, if enough OR time saved can be accrued within a given day, and the OR is running at full capacity throughout the year, and there is a queue for procedures or opportunity to expand the demand for OR procedures, hospitals may be able to increase annual procedural throughput and decrease surgical waiting times.

The impact of clinical sequelae of residual blockade is a relatively under-researched area. It is important to note that available literature were insufficient for identifying where excess hospital resource use is incurred for rarer but more serious respiratory outcomes which could potentially occur (e.g., post-operative myocardial infarction in cardiovascular disease patients, aspiration pneumonia in emergency cases where patients are unable to be operated on with an empty stomach). Further research is needed to better understand the degree to which these rarer clinical events are linked to residual block and potentially avoidable with sugammadex use. It is evident, however, that both residual blockade and its sequelae are very common in the OR and PACU, with an estimated risk of residual blockade at extubation of 60% with neostigmine use as reported herein [6, 19, 20].

## Conclusions

Sugammadex can potentially reduce the risk of RNMB, and/or enhance operating room efficiency, relative to use of neostigmine for routine reversal of neuromuscular block. In clinical practice within Canada, for the majority of patients currently managed with moderate neuromuscular block, the principal impact of using sugammadex instead of neostigmine is likely to be a reduction in the risk of residual blockade and associated complications. For patients maintained at a deep level of block to the end of the procedure, sugammadex is likely to both enhance OR efficiency and reduce complications of residual block. Where OR efficiency gains occur, potential benefits of sugammadex may include reduced procedural cancellations due to OR time over-run, avoided staff over-time and opportunity to evaluate if procedural throughput may be increased.

## Additional file

Additional file 1: Additional details on estimation of selected model input values. (DOC 96 kb)
