# Supplementary material for: A discrete event simulation model of clinical and operating room efficiency outcomes of sugammadex versus neostigmine for neuromuscular block reversal in Canada
Source: BMC Anesthesiol. 2016 Nov 16;16:114. doi: 10.1186/s12871-016-0281-3 (PMC5112647; doi:10.1186/s12871-016-0281-3)
Supplement: Additional file 1: — Additional details on estimation of selected model input values. (DOC 96 kb) [file 12871_2016_281_MOESM1_ESM.doc]

**Additional File 1**

***Risk of Residual Blockade at Extubation***

As described in the main text, studies describing the risk of residual blockade at extubation exclusively with rocuronium or vecuronium and neostigmine use, when patients are not required to have verification of full neuromuscular recovery (TOF ratio ≤ 0.9) prior to extubation in the OR, were meta-analyzed using a random effects model. Results from the three eligible studies are reported in Table A1.

**Table A1.** Risk of residual neuromuscular blockade (TOF <0.9) at extubation

| **Study** | **N** | **Residual Block (n)** | **Residual Block (%)** |
| --- | --- | --- | --- |
| Murphy et al. [1] | 120 | 105 | 88% |
| Sabo et al. [2] | 43 | 26 | 60% |
| Kotake et al. [3] | 109 | 26 | 24% |
| Random effects meta-analysis | 272 | 157 | 60% |

NMBA = neuromuscular blocking agent; TOF = train-of-four

***Risk of Hypoxemia With and Without Residual Blockade***

Hypoxemia (low oxygen levels) has been documented to occur more frequently in patients with residual blockade and likely results from impairment of the hypoxic ventilatory response and respiratory muscle strength[4]. It has been defined as "mild" when patients have hemoglobin oxygen (O_2_) saturations (Spo_2_) of 93-90% and "severe" when Spo_2_ levels fall below 90%[4].

A search of the PubMed database on July 10, 2015 using terms of [hypoxia or hypoxemia] AND [residual blockade or residual block or curarization] did not identify any studies reporting a risk of hypoxemia when residual blockade was assessed following extubation. Three studies were identified reporting data on hypoxemia when residual blockade (defined as TOF < 0.9), following administration of intermediate-acting NMBAs, was assessed in the post-anesthesia care unit (PACU)[5–7]. In the first study, all patients were administered Rocuronium (n = 114), and the risk of hypoxemia on PACU arrival was 50.9% in the 'residual blockade' group compared to 28.1% in the 'non-residual blockade group'. However the study design was more experimental in nature in that quantitative neuromuscular monitoring was performed, and all patients without residual blockade were extubated with a TOF ratio of exactly 1.0[7]. Because patients in clinical practice without residual blockade may have TOF ratios ranging from 0.9 to greater than 1.0, it is not clear how generalizable the data for this group are to actual practice. Also, at least 1 patient with a TOF ratio at extubation equal to 0.9 was included in the group evaluated as having residual blockade. A second study included a large sample of patients (n = 624), but only evaluated risks of severe hypoxemia (Spo2 < 90%), which would underestimate overall risk[5]. The third study evaluated patients (n =202) managed according to usual clinical practice, and reported a risk of hypoxemia that was higher, upon PACU admission, for patients with residual blockade (30%) than those without residual blockade (5%)[6]. Patients were generally well balanced on other characteristics between the residual blockade and no residual blockade groups. Based on the strengths and limitations of each analysis, data from the third study were therefore used within the model. It would have been preferable to have had data on the risk of hypoxemia with residual blockade assessed at extubation (as was the point of assessment in the clinical trial evaluating prevention of residual blockade with sugammadex versus neostigmine[2]) rather than in the PACU, however, in the absence of this information, default data were directly extrapolated to cases of residual blockade at extubation (Table A2).

**Table A2.** Risk of post-operative hypoxemia with and without residual blockade

| **Residual blockade (TOF < 0.9) assessed at PACU arrival** | **N (Patients)** | **n (Hypoxemia)** | **% (Hypoxemia)** |
| --- | --- | --- | --- |
| Residual blockade | 61 | 18 | 29.5% |
| No residual blockade | 141 | 7 | 5.0% |

PACU = post-anesthesia care unit; TOF = train-of-four

***Risk of Upper Airway Obstruction With and Without Residual Blockade***

Upper airway obstruction has been defined as a ratio of maximal expiratory flow and maximal inspiratory flow at 50% of vital capacity (MEF50/MIF50) of greater than 1 and can be detected by spirometry[8]. It can occur during anesthesia and neuromuscular block through decrease in muscle tone associated with loss of wakefulness, and drug-induced inhibition of upper airway neural and muscle activity and suppression of protective arousal responses[9].

A search of the PubMed database using terms of [obstruction] AND [residual blockade or residual block or curarization] identified one study[6], describing the association between residual blockade (defined as TOF < 0.9) and post-operative upper airway obstruction in patients receiving intermediate acting NMBAs. This study of patients in the PACU found that cases of upper airway obstruction requiring intervention were significantly more likely in patients with residual blockade versus those without. However, upper airway obstruction events were based on those detected clinically and for which an intervention (jaw thrust, oral airway or nasal airway) was undertaken and is likely to under-estimate the actual occurrence of upper airway obstruction physiologically in clinical practice. In the absence of patient studies in the operative setting, to obtain an estimate of the latter, experimental studies of residual blockade induced in healthy volunteers were reviewed. One study was identified using an intermediate acting NMBA, which assessed upper airway obstruction as a ratio of MEF50/MIF50[10]. In that study, 12 healthy male volunteers were administered Rocuronium and the incidence of upper airway obstruction was evaluated at various TOF ratios (Table A3). In the analysis results, these were reported as TOF ratios (mean ± SD) of 0.5 ± 0.16, 0.83 ± 0.06 and 1.02 ± 0.01. In the absence of more refined estimates, the observed incidences of upper airway obstruction at these TOF levels were presumed to correspond to TOF ranges of ≤ 0.70, > 0.70-< 0.90 and ≥ 0.90 in applying them to post-operative TOF data following extubation from the Sugammadex Protocol 334 trial (Rocuronium + Neostigmine arm)[11] to estimate an overall risk of upper airway obstruction in patients with vs. without residual blockade (Table A4).

**Table A3.** Risk of upper airway obstruction by TOF range

| **Reported TOF Ratio (mean ± SD)** | **Converted TOF Range** | **N** | **n (Upper Airway Obstruction)** | **% (Upper Airway Obstruction)** |
| --- | --- | --- | --- | --- |
| 0.5 ± 0.16 | ≤ 0.70 | 12 | 8 | 66.7% |
| 0.83 ± 0.06 | > 0.70-< 0.90 | 12 | 4 | 33.3% |
| 1.02 ± 0.01 | ≥ 0.90 | 12 | 1 | 8.3% |

SD = standard deviation; TOF = train-of-four

**Table A4.** Risk of upper airway obstruction with vs. without residual blockade (TOF ≥ 0.9) following extubation

| **TOF Ratio** | **n**[2] | **Estimated % With Upper Airway Obstruction**[10] | **Upper Airway Obstruction Without Residual Blockade (%)** | **Upper Airway Obstruction With Residual Blockade (%)** |
| --- | --- | --- | --- | --- |
| ≤ 0.7 | 15 | 66.7% |  |  |
| > 0.7-<0.9 | 11 | 33.3% |  |  |
| ≥ 0.9 | 17 | 8.3% |  |  |
| Total | 43 | 35.1% | 8.3% | 52.6% |

TOF = train-of-four

***Model Literature Review Criteria***

Systematic literature searches of the PubMed database for model input values (up to August 21, 2015) are listed below. All searches were limited to English language citations.

In the case of operating room scheduling and staffing parameters (e.g., time to start of OR day, turnover times, time for OR clean-up, procedure duration, OR staff eligible for over-time pay), values would expected to be highly variable across operating rooms and institutions. With respect to scheduling, available publications reporting data often reflect examples where efficiency was less than desired and improvements were sought. Rather than conducting a formal meta-analysis across publications, data from available sources deemed as reasonable and credible by OR experts were included within the model and values were varied in reported sensitivity analyses.

| **Search Number (Input Variable)** | **Search Terms** |
| --- | --- |
| Search 1 (Risk of residual  neuromuscular blockade) | (residual blockade or residual block or curarization) and (rocuronium or vecuronium) |
| Search 2 (Risk of hypoxemia) | (hypoxia or hypoxemia) AND (residual blockade or residual block or curarization) |
| Search 3 (Risk of upper airway  obstruction) | (obstruction) AND (residual blockade or residual block or curarization) |
| Search 4 (Sugammadex impact on  OR time and residual  neuromuscular blockade) | (sugammadex AND trial) |

References

1. Murphy GS, Szokol JW, Marymont JH, Franklin M, Avram MJ, Vender JS (2005) Residual paralysis at the time of tracheal extubation. Anesth Analg 100: 1840-1845.

2. Sabo D, Jones RK, Berry J, Sloan T, Chen JY, Morte JB, Groudine S (2011) Residual neuromuscular blockade at extubation: A randomized comparison

of sugammadex and neostigmine reversal of rocuronium-induced blockade in patients undergoing abdominal surgery. J Anesthes Clin Res 2: 140.

3. Kotake Y, Ochiai R, Suzuki T, Ogawa S, Takagi S, Ozaki M, Nakatsuka I, Takeda J (2013) Reversal with sugammadex in the absence of monitoring did not preclude residual neuromuscular block. Anesth Analg 117: 345-351.

4. Murphy GS, Szokol JW, Marymont JH, Greenberg SB, Avram MJ, Vender JS (2008) Residual neuromuscular blockade and critical respiratory events in the postanesthesia care unit. Anesth Analg 107: 130-137.

5. Cammu GV, Smet V, De Jongh K, Vandeput D (2012) A prospective, observational study comparing postoperative residual curarisation and early adverse respiratory events in patients reversed with neostigmine or sugammadex or after apparent spontaneous recovery. Anaesth Intensive Care 40: 999-1006.

6. Norton M, Xara D, Parente D, Barbosa M, Abelha FJ (2013) Residual neuromuscular block as a risk factor for critical respiratory events in the post anesthesia care unit. Rev Esp Anestesiol Reanim 60: 190-196.

7. Sauer M, Stahn A, Soltesz S, Noeldge-Schomburg G, Mencke T (2011) The influence of residual neuromuscular block on the incidence of critical respiratory events. A randomised, prospective, placebo-controlled trial. Eur J Anaesthesiol 28: 842-848.

8. Eikermann M, Blobner M, Groeben H, Rex C, Grote T, Neuhauser M, Beiderlinden M, Peters J (2006) Postoperative upper airway obstruction after recovery of the train of four ratio of the adductor pollicis muscle from neuromuscular blockade. Anesth Analg 102: 937-942.

9. Hillman DR, Platt PR, Eastwood PR (2003) The upper airway during anaesthesia. Br J Anaesth 91: 31-39.

10. Eikermann M, Groeben H, Husing J, Peters J (2003) Accelerometry of adductor pollicis muscle predicts recovery of respiratory function from neuromuscular blockade. Anesthesiology 98: 1333-1337.

11. (2009) A multi-center, randomized, parallel group, comparative, active controlled, safety assessor blinded, anesthesiologist-TOF-Watch® SX blinded trial comparing T4/T1 ratio at time of tracheal extubation using 4 mg.kg-1 sugammadex administered at 1-2 PTCs or better after the last dose of rocuronium bromide to 50 ?g.kg-1 neostigmine administered as per standard of care in adult subjects undergoing elective open abdominal procedures requiring neuromuscular blockade reversal. Clinical Trial Report on Protocol 19.4.334. Merck & Co., Inc <Data on file>.
